# Supplementary material for: Association between oral microbiome and seven types of cancers in East Asian population: a two-sample Mendelian randomization analysis
Source: Front Mol Biosci. 2023 Nov 21;10:1327893. doi: 10.3389/fmolb.2023.1327893 (PMC10702768; doi:10.3389/fmolb.2023.1327893)
Supplement: Supplementary file 1 [file DataSheet1.ZIP › Supplementary Materials/MR plots of saliva/saliva═╝/Pancreatic cancer/pheno.773_to_pancreatic cancer_leave_one_out.pdf]

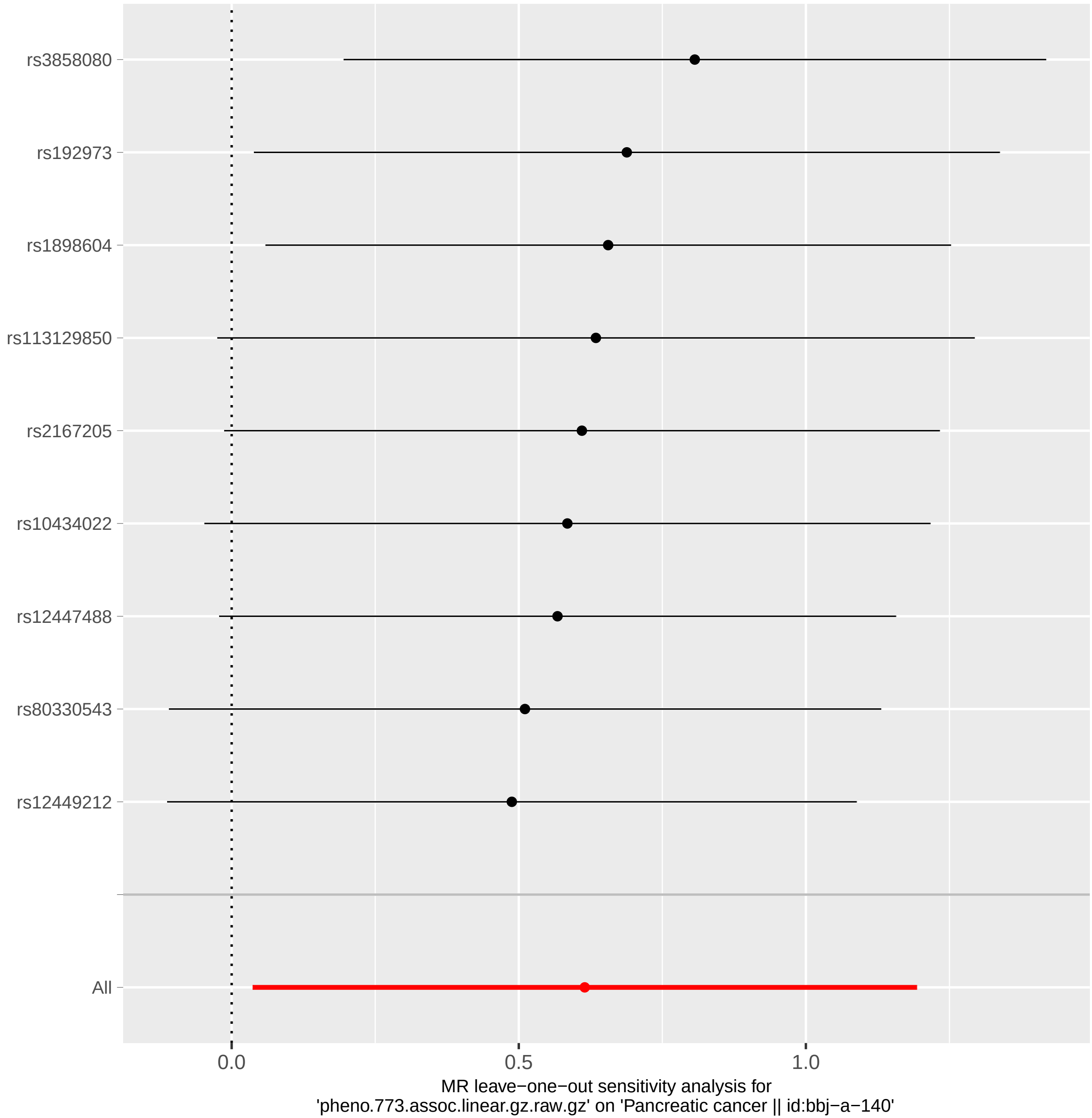
rs3858080
rs192973
rs1898604
rs113129850
rs2167205
rs10434022
rs12447488
rs80330543
rs12449212
All
0.0
0.5
1.0
MR leave−one−out sensitivity analysis for
'pheno.773.assoc.linear.gz.raw.gz' on 'Pancreatic cancer || id:bbj−a−140'
